# Differential Requirement for *irf8* in Formation of Embryonic and Adult Macrophages in Zebrafish

**DOI:** 10.1371/journal.pone.0117513

**Published:** 2015-01-23

**Authors:** Celia E. Shiau, Zoe Kaufman, Ana M. Meireles, William S. Talbot

**Affiliations:** Department of Developmental Biology, Stanford University School of Medicine, Stanford, California, United States of America; Hong Kong University of Science and Technology, CHINA

## Abstract

Interferon regulatory factor 8 (Irf8) is critical for mammalian macrophage development and innate immunity, but its role in teleost myelopoiesis remains incompletely understood. In particular, genetic tools to analyze the role of Irf8 in zebrafish macrophage development at larval and adult stages are lacking. We generated *irf8* null mutants in zebrafish using TALEN-mediated targeting. Our analysis defines different requirements for *irf8* at different stages. *irf8* is required for formation of all macrophages during primitive and transient definitive hematopoiesis, but not during adult-phase definitive hematopoiesis starting at 5-6 days postfertilization. At early stages, *irf8* mutants have excess neutrophils and excess cell death in *pu.1*-expressing myeloid cells. Macrophage fates were recovered in *irf8* mutants after wildtype *irf8* expression in neutrophil and macrophage lineages, suggesting that *irf8* regulates macrophage specification and survival. In juvenile *irf8* mutant fish, mature macrophages are present, but at numbers significantly reduced compared to wildtype, indicating an ongoing requirement for *irf8* after embryogenesis. As development progresses, tissue macrophages become apparent in zebrafish *irf8* mutants, with the possible exception of microglia. Our study defines distinct requirement for *irf8* in myelopoiesis before and after transition to the adult hematopoietic system.

## Introduction

Myeloid cells form the innate immune system that provides the immediate response to protect the host after infection and injury. Macrophages, monocytes, and granulocytes (including neutrophils) are major myeloid cell types [1–4]. The proper formation of these myeloid cells during development and their continuous replenishment throughout life are essential to sustain the function of the immune system. As in mammals, hematopoiesis in zebrafish occurs in several waves [1, 5, 6]. This process begins with the primitive wave in the anterior lateral plate mesoderm of the zebrafish embryo, then transitions to the transient definitive wave in the posterior blood island (PBI), which later becomes the caudal hematopoietic tissue (CHT). Subsequently, definitive blood development takes place in the CHT and the zebrafish aorta-gonad-mesonephros (AGM) analog. At later stages, definitive hematopoiesis moves to the pronephric kidney, which is the site of adult hematopoiesis [5, 6]. More is known about the development of hematopoietic stem cells (HSCs) into different myeloid fates in the definitive wave than in the earlier waves of blood formation [2, 3, 6, 7], although the mechanisms are not fully understood in either the early or late waves.

A major determinant of macrophage/monocyte fate during hematopoiesis is the transcription factor IRF8, a member of the interferon regulatory factor (IRF) family. IRF proteins contain a conserved N-terminal DNA binding domain that recognizes the interferon consensus sequence, and they regulate transcription of interferon genes during immune response [8]. In mammals, IRF8 is critical for myeloid development. IRF8 deficiency in human and mouse leads to a significant reduction of macrophage/monocyte development but an expansion of neutrophils and undifferentiated hematopoietic progenitor cells, reminiscent of myeloid leukemia [9–12]. In mouse, IRF8 is strongly expressed in mononuclear phagocytes (macrophages, monocytes, and dendritic cells)[3, 13], and regulates differentiation of these phagocytes and granulocyte-macrophage progenitors [9, 10, 14]. In human, missense mutations disrupting transcriptional activity of IRF8 are linked with immunodeficiencies that severely reduce the numbers of dendritic cells and monocytes [11], indicating its role in human myeloid development. Furthermore, IRF8 is transcriptionally repressed in many acute and chronic myeloid leukemia (AML and CML, respectively) patients [15, 16], suggesting a link between IRF8 activity and these diseases. In zebrafish, transient knockdown of *irf8* by translation- and splice- blocking morpholinos eliminated the embryonic macrophage population and expanded the neutrophil population [17], but mutations in zebrafish *irf8* have not been available to enable long-term studies. Thus, IRF8 plays key roles in maintaining normal production of myeloid cell types, but the nature of its function in specification and maintenance of myeloid cells at different times in development are not understood, and the extent of its functional conservation across species remains to be fully explored.

To investigate the function of *irf8* at different stages of development to early adulthood in zebrafish, we created *irf8* null mutations. *irf8* mutants are devoid of macrophages during embryogenesis, but have an increase of neutrophils and immature or apoptotic myeloid cells. As larval development progresses, some macrophages are present in the mutants, indicating that macrophages at different stages have distinct requirements for Irf8 function. The late-emerging macrophages in *irf8* mutants are likely derived from different progenitors from the embryonic macrophages (HSCs versus early myeloid), which coincides with the differential dependence on *irf8*. Our study provides new insights into the role of *irf8* in myeloid cell fate regulation in zebrafish before and after transition to the adult hematopoietic system.

## Results

### *irf8* null mutants lack microglia during development and are viable

**Fig 1:**
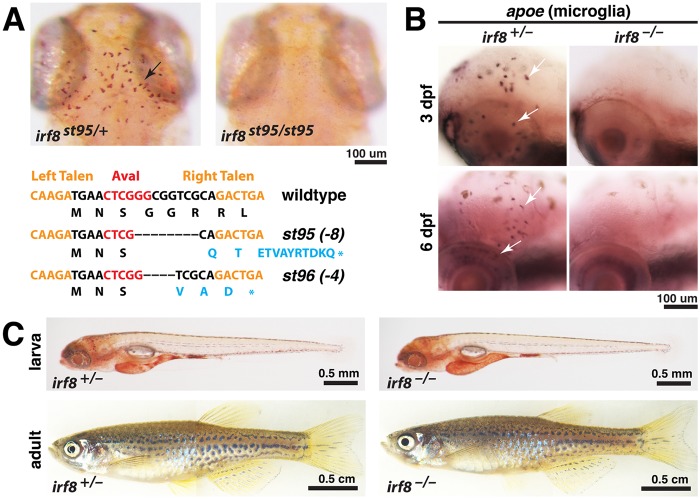
TALEN-induced *irf8* mutations *st95* and *st96* eliminate embryonic microglia but allow survival to adulthood. (A) TALE nucleases target region near *irf8* translational start site, creating frameshift mutations *st95* and *st96*, which introduced premature stop codons as shown (bottom). Top panels show representative neutral red staining for microglia in *irf8* ^*st95/st95*^ mutant that lacks all microglia, compared with a heterozygous sibling that has a wildtype microglial population (arrow). (B) Analysis of *apoe* RNA expression by in situ hybridization shows presence of microglia in *irf8* sibling but no microglia in *irf8* mutants at 3 and 6 dpf. (C) Images of heterozygous and homozygous mutant larvae at 5 dpf, showing that the mutants have normal overall morphology. Images of the whole adult zebrafish were compiled from two tiled images of the same fish. At 3 months of age, the *irf8* mutant zebrafish grew to a similar size as its sibling. All images represent the *st95* allele. Scale bars are shown below each set of panels or for each individual panel.

Morpholino knockdown studies have indicated that *irf8* plays a role in macrophage development in zebrafish [17], but mutations in the zebrafish *irf8* gene have not been available to allow analysis of a complete loss of *irf8* function, or analysis at later stages. Furthermore, excess immature myeloid cells were observed in Irf8 mouse mutants but not reported in the zebrafish morpholino studies [17], so the degree of conservation of Irf8 function between zebrafish and mammals is not fully understood. To construct loss-of-function mutations in *irf8*, we used TALEN-mediated targeting to generate two new mutant alleles, *irf8* ^*st95*^ and *irf8* ^*st96*^ (Fig. 1A). Both alleles have frameshift mutations just 3’ to the translational initiation codon, and both are predicted null mutations (Fig. 1A). The zebrafish genome contains only one ortholog of mammalian Irf8, and therefore these mutations are predicted to eliminate all functions of Irf8 in zebrafish.

To characterize the phenotypes of *irf8* ^*st95*^ and *irf8* ^*st96*^ mutants, we examined microglia, the brain-resident macrophages previously shown to be disrupted by splice- and translation- blocking morpholinos against *irf8* [17]. Homozygous *irf8* ^*st95/st95*^ and *irf8* ^*st96/st96*^ mutants, and transheterozygous *irf8* ^*st95/st96*^ mutants all lacked microglia at 3–6 dpf, as determined by neutral red staining [19] and expression of *apoe* [20–22](Fig. 1A and B, and data not shown). We have focused our analysis on *irf8* ^*st95*^, and the *irf8* ^*-/-*^ mutants pictured in the figures are *irf8* ^*st95/st95*^ homozygotes, unless noted otherwise.

Despite the loss of microglial cells during the embryonic and larval stages, *irf8* ^*-/-*^ mutants appeared morphologically normal as early larvae (Fig. 1C). Furthermore most *irf8* ^*-/-*^ mutants were viable as adults at approximately 3 months postfertilization (Fig. 1C), although their survival rate was reduced compared to heterozygous and wildtype siblings (S1 Fig.).

### *irf8* mutants have no macrophages but have excess neutrophils during embryogenesis

**Fig 2:**
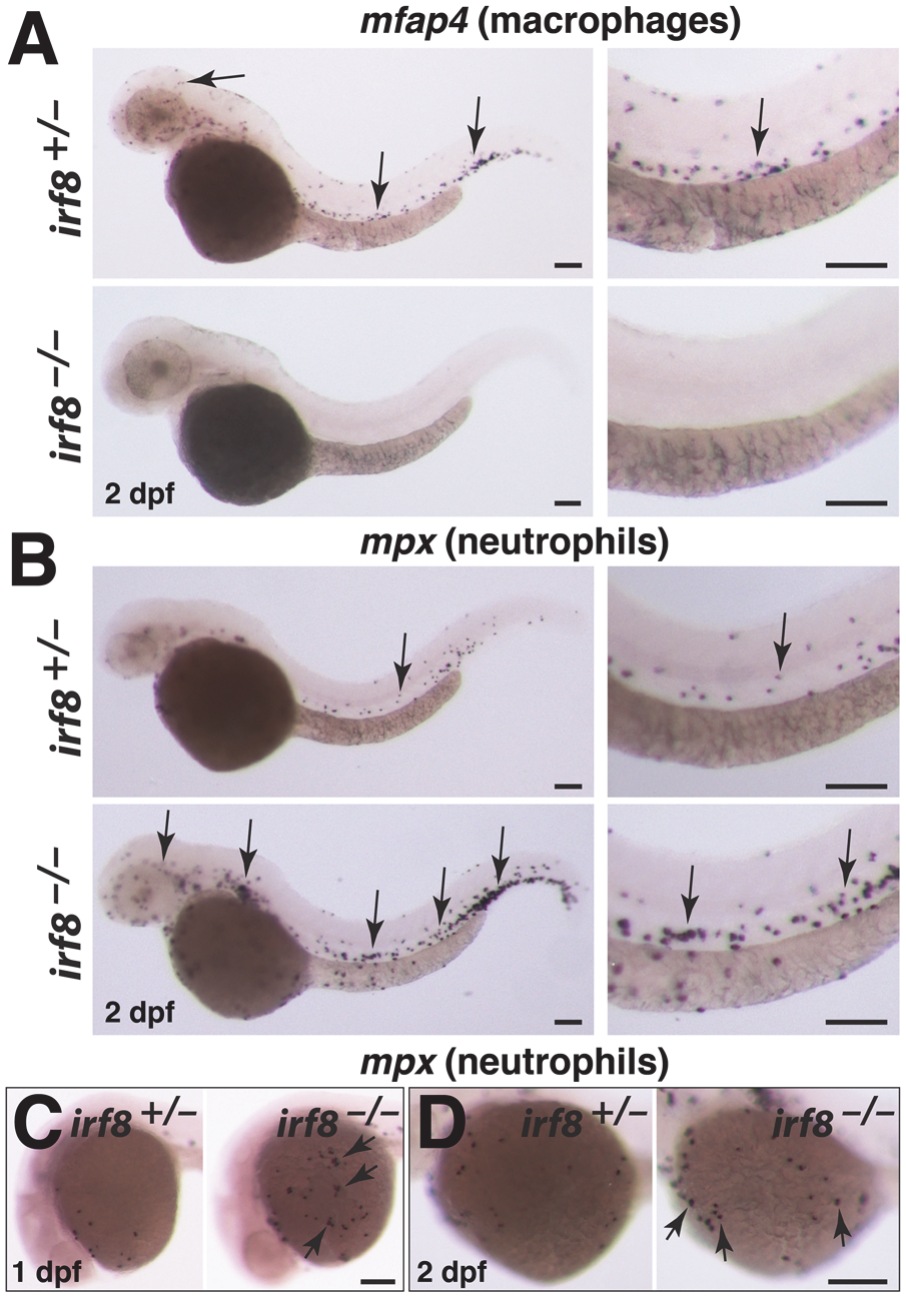
*irf8* mutant embryos have no macrophages but produce excessive neutrophils. (A) *mfap4* RNA expression at 2 dpf shows a complete loss of macrophages in *irf8* mutant but abundant macrophages in heterozygous sibling (arrows). (B) *mpx* RNA expression at 2 dpf shows an overproduction of neutrophils in *irf8* mutants (arrows, bottom panels) compared with the sibling (arrow, top panels). Right column in A and B shows a higher magnification image of the trunk region from the same embryos depicted in the left column. (C) Neutrophils can first be detected on the yolk sac by 1 dpf. *irf8* mutants have many yolk sac neutrophils (arrows) compared with sibling. (D) At 2 dpf, *irf8* mutants continue to have more neutrophils on the yolk sac (arrows). Overall, *irf8* mutants appear to have more neutrophils throughout the body. All scale bars are 100 um.

To further characterize the *irf8* mutants, we examined the early development of myeloid cells at embryonic stages. In zebrafish, adaptive immunity begins after 3–4 weeks postfertilization [23], and macrophages and neutrophils are the main functional leukocytes in the embryo [18]. We assessed the distribution of macrophages and neutrophils by examining the cell-type specific markers *mfap4* and *mpx*, respectively. In comparison to wildtype and heterozygous siblings, *irf8* ^*-/-*^ mutants developed no *mfap4*-expressing macrophages (Fig. 2A), and had a large expansion of *mpx*-expressing neutrophils (Fig. 2B and C). The increase in neutrophils was evident throughout the embryo, and at stages as early as 1 dpf (Fig. 2B–D). Our genetic analysis validates the embryonic phenotypes observed previously in *irf8* morphants [17]. In addition, the finding that *irf8* null mutants are viable as adults allows the analysis of *irf8* function into adulthood.

### Partial recovery of macrophages in *irf8* mutants coincides with presumptive onset of hematopoiesis in the kidney

**Fig 3:**
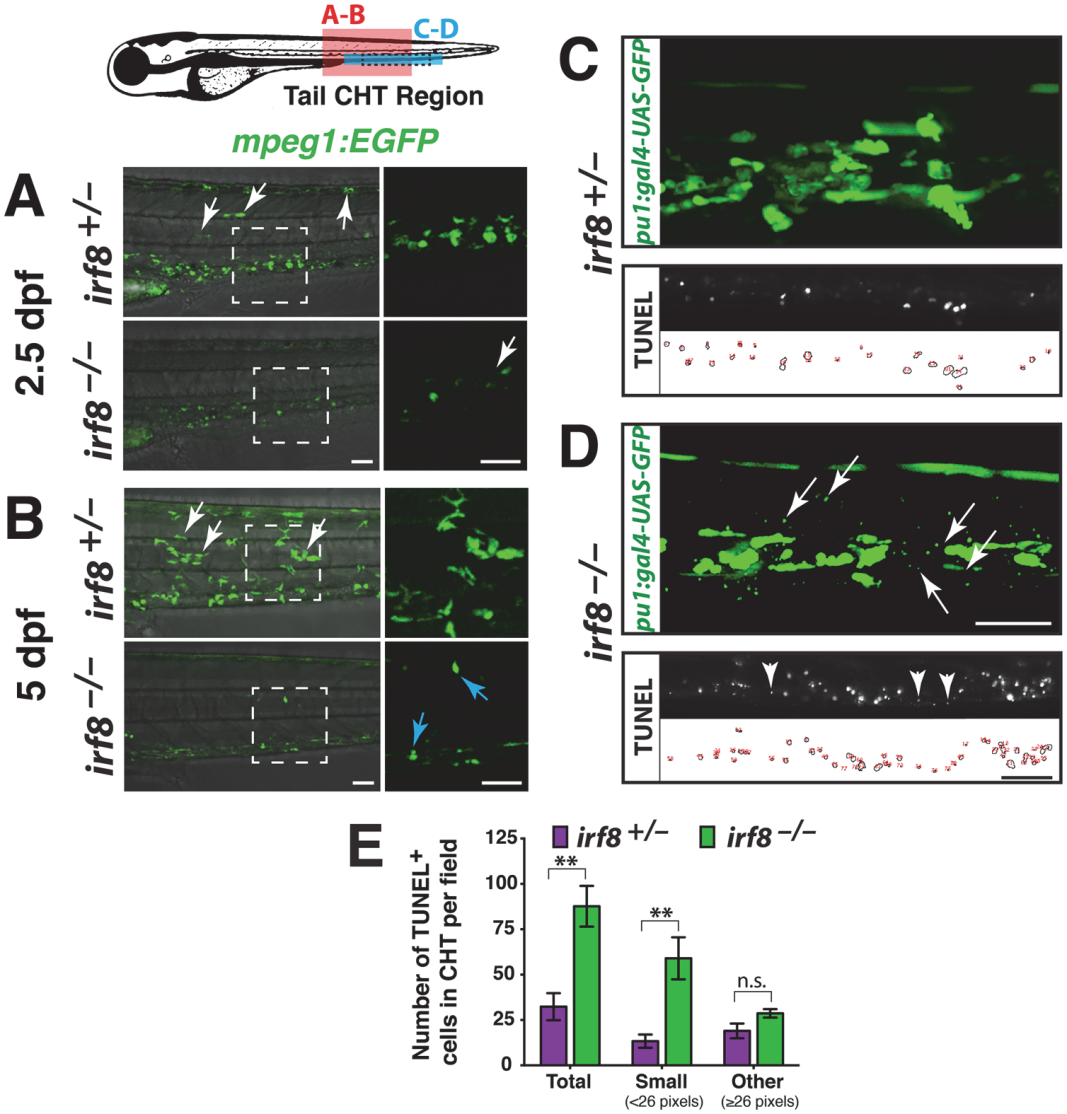
*irf8* mutants have immature myeloid cells and excess myeloid cell death during development in the CHT. Diagram of a zebrafish larva showing the regions of analysis in the CHT: red box, region in A and B; blue box, region in C and D; and dotted lines in blue box indicate area of TUNEL quantification in E. (A-B) Lateral view. Right panels, higher magnification of the dotted box shown on the left. Analysis at 2.5 dpf (A) and 5 dpf (B) shows that siblings have many macrophages strongly expressing *mpeg1*:*EGFP*; these cells have elaborate processes and complex morphologies, and some have migrated into other tissues (white arrows). By contrast, *irf8* mutants have cells weakly expressing *mpeg1*:*EGFP* that appear immature and different from macrophages in siblings. A few strongly expressing cells are first detected in mutants at 5 dpf (B, blue arrows), indicating recovery of a few macrophages. (C-D) Early myeloid reporter *pu*.*1*:*gal4-UAS-GFP* at 5 dpf shows abnormally small cellular specks restricted to the CHT in all mutants (D, arrows, n = 9/9 at 3 and 5 dpf) but not in the siblings (C, n = 5/5 at 3 and 5 dpf). Bottom, TUNEL labeling of apoptotic cells at 5 dpf. The small *pu*.*1* reporter expressing cellular specks in the CHT are similar in size and appearance to small-sized dying cells labeled by TUNEL, which are shown in another 5 dpf stage-matched *irf8* mutant larva (compare arrows in top panel with arrowheads in bottom panel from different larvae). TUNEL labeling (middle) and area traces of the TUNEL+ nuclei (bottom) are shown for each genotype. (E) Quantification of TUNEL assay as represented in C-D shows a significant increase in total dying cells in the CHT of *irf8* mutants (n = 3) compared with siblings (n = 6; p = 0.0039). This is largely accounted for by a significant increase in very small-sized TUNEL+ cells measuring less than 26 pixels in area (p = 0.0017). No significant difference was found in larger TUNEL+ cells (≥ 26 pixels in area, p = 0.16). Error bars represent S.E.M. Statistical significance was determined by two-tailed Student’s t-test. **, p < 0.01; *, p < 0.05; n.s., not significant; CHT, caudal hematopoietic tissue. All scale bars are 50 um and are the same for each set of panels.

**Fig 4:**
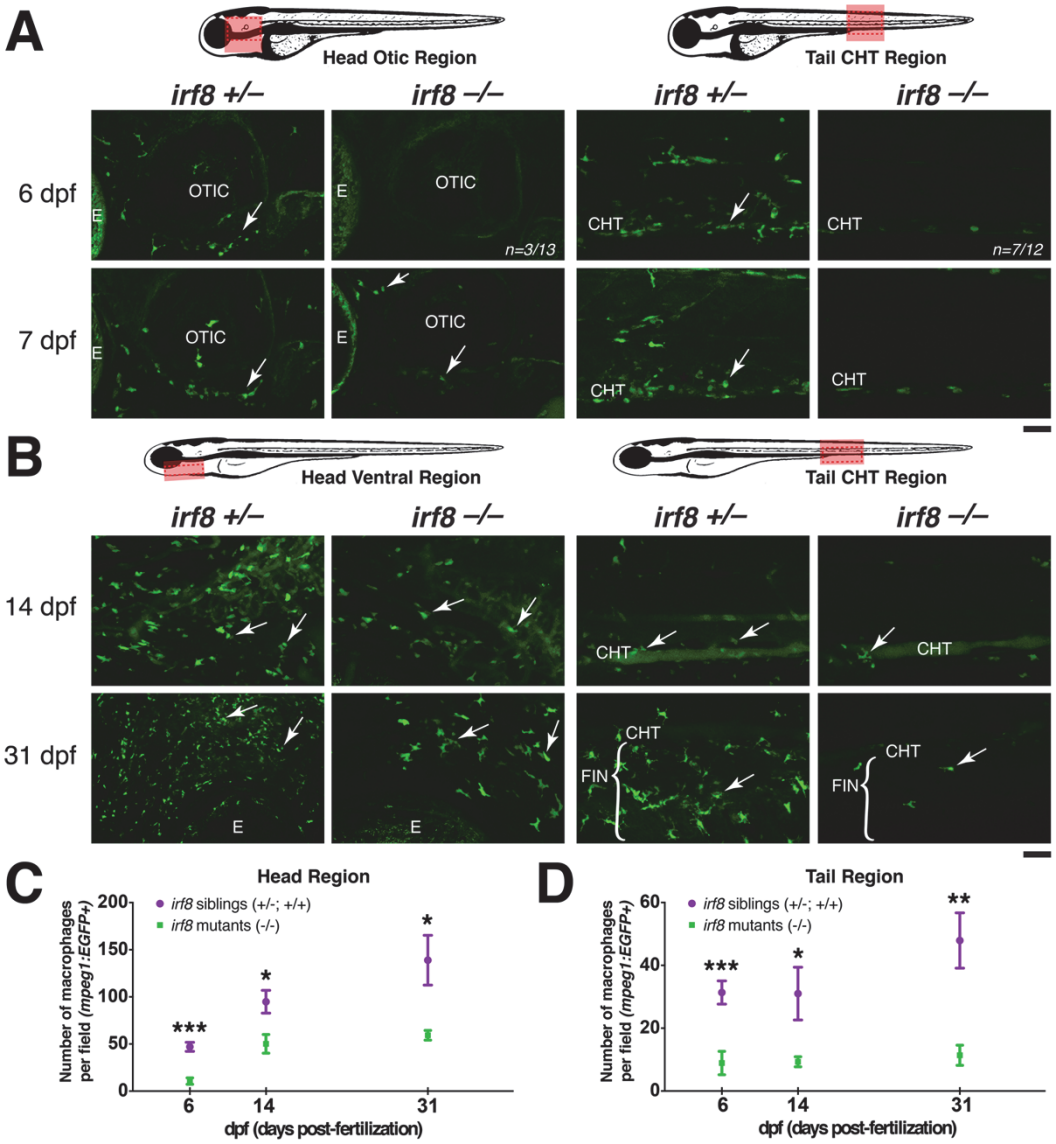
Partial recovery of macrophages in *irf8* mutants begins by ~7 dpf in the head region. Diagrams of zebrafish showing region of quantification (pink box), which was taken using the same magnification and field size of view at all stages. The field of view in older stages covers a relatively smaller region of the head because the fish are larger (comparing B with A). Red dotted box shows the region of the fluorescent images. (A) Expression of macrophage reporter *mpeg1*:*EGFP* at 6 and 7 dpf. Arrows show macrophages. At 6 dpf, most *irf8* mutants have no macrophages in the CHT (n = 7/12), while most have a few macrophages in the head (n = 10/13); image shows the head region of a mutant at 6 dpf with no macrophages. Panels show the tail CHT region of the *irf8* mutants at 6 and 7 dpf with no macrophages; a few autofluorescent pigment cells are present after PTU treatment. (B) Distribution of macrophages expressing *mpeg1*:*EGFP* at 14 dpf and 31 dpf. Arrows point to macrophages. (C) Quantification of the number of macrophages per field in the head region over time (pink box in left diagram). (D) Quantification of the number of macrophages per field in the tail region over time (pink box in right diagram). At 6 dpf, n = 20 for siblings and n = 13 for mutants; at 14 dpf, n = 8 for siblings and n = 7 for mutants; at 31 dpf, n = 7 for siblings and n = 5 for mutants. Statistical significance was determined by two-tailed Student’s t-test. Error bars represent S.E.M. ***, p < 0.001; **, p < 0.01; *, p < 0.05; CHT, caudal hematopoietic tissue; E, eye. All scale bars are 50 um. *irf8* ^*-/-*^ in this figure represents trans-heterozygous *irf8* ^*st95/st96*^ mutants.

To address the possible roles of *irf8* in macrophage development at later stages, we used *Tg (mpeg1*: *EGFP)* to examine GFP-labeled macrophages at stages up to 31 dpf in *irf8* ^*-/-*^ mutants and their siblings. These macrophages were examined in live fish up to 1 week old (7 dpf) and after fixation at later stages (Figs. 3 and 4). We analyzed two regions where macrophages are normally widespread: first, the caudal hematopoietic tissue (CHT) in the ventral mesenchyme of the tail, which is the major location of new blood formation at larval stages; and second, the otic or ventral area of the head that is proximal to the pronephric kidney, the location of adult hematopoiesis (Figs. 3 and 4).

Although there was a lack of mature *mfap4*-expressing macrophages at embryonic stages (Fig. 2), we observed small cells that weakly expressed *mpeg1*:*EGFP* at 2.5 dpf in the mutants. These cells were restricted to blood-forming regions in the tail—they did not disperse to other tissues, in contrast to wildtype macrophages (Fig. 3A). These cells may be immature myeloid cells, based on their location and weak *mpeg1* reporter expression, lack of detectable *mfap4* expression (Fig. 2), and the previous finding that Irf8 knockout mice have unchecked proliferation of immature myeloid cells [9, 12, 24]. Alternatively, these small cells in the mutants may be dying myeloid cells or cell debris.

By 5 dpf, a few strongly labeled *mpeg1*-expressing macrophages were present in some homozygous *irf8* mutants, but other mutants appeared to have no macrophages (Figs. 3B and 4). At this time, the mutants also showed significantly increased cell death in the CHT region (Fig. 3C and D), evidenced by an increase of very small TUNEL-positive cells (Fig. 3E). In addition, we detected aberrant, small-sized or rounded cells and fragments expressing the *pu*.*1* reporter transgene in all mutants analyzed but not in the siblings (Fig. 3C–E), which is consistent with myeloid precursor cell death in *irf8* mutants. In summary, *irf8* mutants have abnormally small cells that weakly express *mpeg1*:*EGFP*, and they have significantly increased cell death in the *pu*.*1*+ myeloid precursors.

By 7dpf, *irf8* mutants contained macrophages strongly expressing *mpeg1*:*EGFP* in the head and thymic region near the otic vesicle (Fig. 4A). These first-appearing macrophages in the *irf8* mutants appear less mature than wildtype, being rounded with few or no processes (Fig. 4A). In the ventral head at 14 dpf and 31 dpf (Fig. 4B), the number of macrophages increased in *irf8* mutants, but it remained less than half of that in wildtype and heterozygous siblings in the head and tail regions (Fig. 4C and D).

In our time course analysis of *mpeg1*:*EGFP* expression at different stages, it was clear that the number of *mpeg1*:*EGFP* expressing macrophages was more strongly reduced in the tail than in the head (Fig. 4C and D). The increased number of macrophages in the ventral head region may reflect proximity to the pronephric kidney, which becomes the predominant site of blood formation starting at about the same time that the first macrophages became evident in *irf8* mutants. The absence of macrophages in *irf8* mutants before 5–6 dpf indicates that *irf8* is a key regulator of all macrophage populations in the embryo and early larva. The recovery that begins in mid-larval stages at ~7 dpf suggests that later precursor cells, such as the hematopoietic stem cells (HSCs) from the kidney marrow, may depend on a combination of *irf8* and other factors. Despite this transition, *irf8* is essential to achieve normal macrophage numbers over the long-term, as the recovery of macrophages in *irf8* mutants is incomplete.

### *irf8* mutants recover macrophages but apparently lack microglia at 31 dpf

The presence of macrophages in the head of *irf8* mutants at 14 dpf and later stages prompted us to determine whether microglia are also present at these stages. We examined microglia in sections of the midbrain at 31 dpf using antibodies against leukocyte marker L-plastin, which labels microglia in the uninjured adult zebrafish brain [25]. At 31 dpf, wildtype fish contained many cells expressing L-plastin with the elaborate morphologies characteristic of microglial cells (S2 Fig.). In *irf8* mutants, we were unable to identify similar cells in the brain parenchyma, but instead observed only a few putative macrophages that were located in the ventricular zone, adjacent to vasculature or in the interstitial space between the brain and the head epithelium (S2 Fig.). These results suggest that microglia are absent in *irf8* mutants at stages up to 31 dpf, several weeks after other macrophages were evident in *irf8* mutants. Our analysis is consistent with previous studies showing that microglia originate from primitive macrophages at early stages but not from later-forming definitive macrophages or monocytes [26, 27].

### *irf8* mutants have myeloid cells and neutrophils competent to become macrophages after expression of wildtype *irf8*

**Fig 5:**
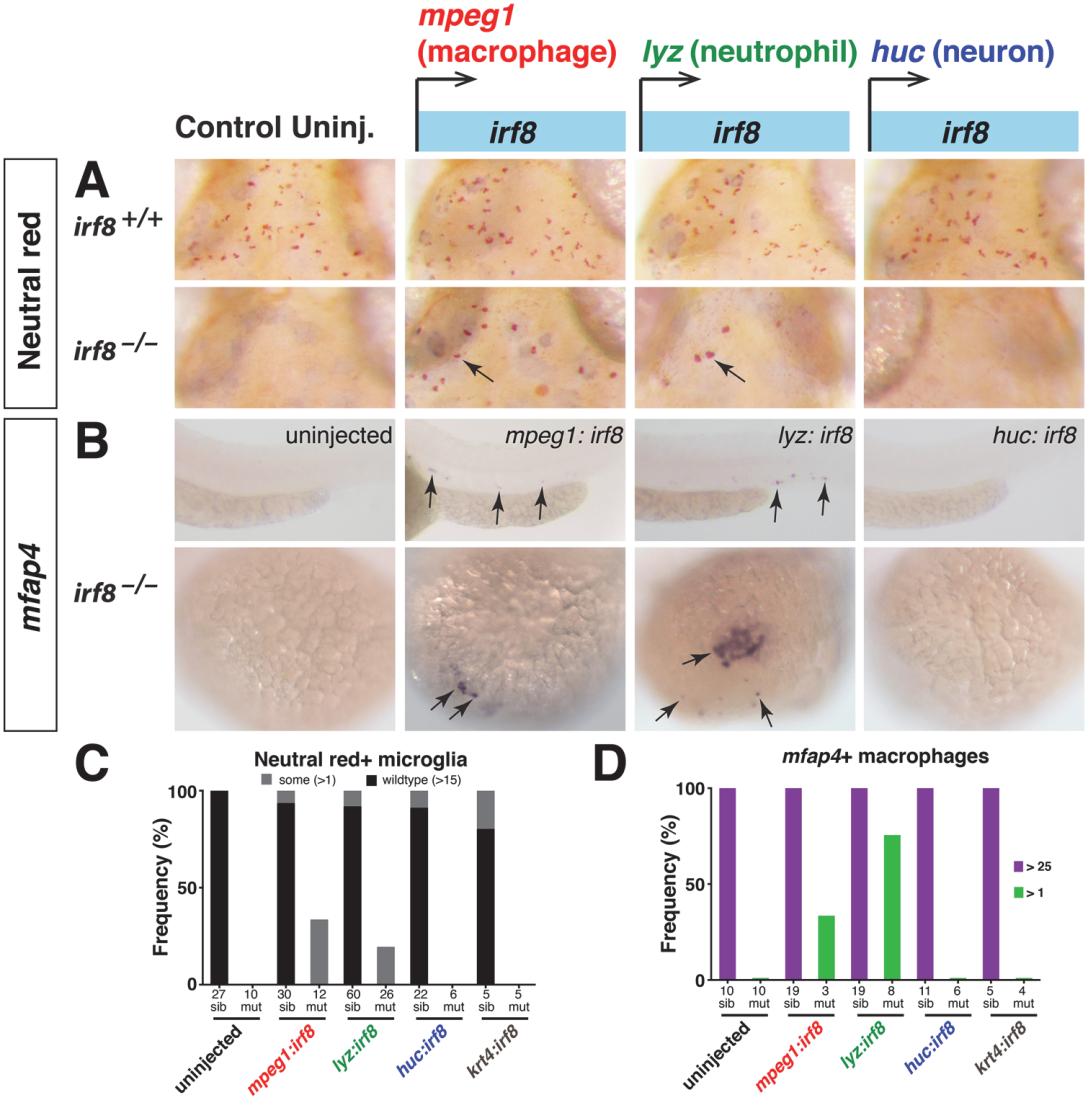
Specific expression of *irf8* in macrophage or neutrophil lineage is sufficient to restore macrophage fates in *irf8* mutants. (A) Neutral red staining for microglia at 4 dpf in *irf8* siblings and mutants in control uninjected conditions or after tol2-mediated expression of *irf8* driven by tissue specific regulatory sequences. Expression of *mpeg1*:*irf8* or *lyz*:*irf8* was sufficient to restore some microglia in *irf8* mutants (arrows), but not expression in the neurons (*huc*) or skin (*krt4*). (B) Analysis of total macrophage population by *mfap4* RNA expression at 2.5 dpf after expression of *irf8* in different tissues. Specific expression of *mpeg1*:*irf8* or *lyz*:*irf8* was also sufficient to restore macrophages on yolk sac and embryo proper (arrows) in *irf8* mutants. (C) Plot showing frequency of microglia rescue. (D) Plot showing frequency of macrophage recovery. Purple denotes >25 macrophages and green shows partial recovery of less than 25 but more than 1. Numbers below bar graphs represent *n*, total number of embryos analyzed. sib, *irf8* ^*+/+*^ and *irf8* ^*+/-*^; mut, *irf8* ^*-/-*^.

A previous *irf8* morpholino study suggested that knockdown of *irf8* caused macrophage progenitors to switch to a neutrophil fate [17], while our data suggested that *irf8* mutants have undifferentiated and dying myeloid cells (Fig. 3) in addition to increased neutrophils. To investigate the possibilities that *irf8* can regulate the specification and survival of macrophage precursors and the fate decisions of neutrophil-macrophage precursors, we sought to restore macrophages in *irf8* mutants by expression of wildtype *irf8* in the macrophage and neutrophil lineages, respectively. Regulatory sequences for *mpeg1*, *lyz*, *huc*, and *krt4*, as previously described [21], were used to express *irf8* in macrophages, neutrophils, neurons, and skin cells, respectively (Fig. 5). These constructs also contain the *cmlc2-GFP* cassette to allow the analysis of animals with successful integration, as detected by strong GFP expression in the heart. We assessed microglial cells by neutral red staining at 4 dpf and the total macrophage population with the marker *mfap4* at 2.5 dpf (Fig. 5). We analyzed the action of the transgenes in embryos from *irf8* heterozygous intercrosses in order to evaluate the effect of *irf8* expression in mutant and wildtype siblings.

When wildtype *irf8* expression was introduced transiently under the control of macrophage-specific regulatory sequences for *mpeg1*, we detected some recovery of macrophages by 2.5 dpf and microglia by 4 dpf in *irf8* mutants, which normally lack all macrophages and microglia at these stages (Fig. 5). The ability to rescue macrophages in *irf8* mutants using the *mpeg1*:*irf8* transgene provides further evidence that the cells weakly expressing the *mpeg1* reporter in *irf8* mutants are immature cells competent to become mature macrophages when wildtype *irf8* is expressed.

To test the other possibility that *irf8* can restore macrophage fate in the expanded neutrophils in *irf8* mutants, we transiently expressed the wildtype *irf8* coding sequence in neutrophils. Strikingly, ectopic expression of *irf8* in neutrophils using *lyz* regulatory sequences led to a partial recovery of microglia and macrophages in the *irf8* ^*-/-*^ mutants (Fig. 5). The rescue of macrophages with the *mpeg1*:*irf8* and *lyz*:*irf8* constructs appeared specific to the macrophage and neutrophil lineages, because the transgenes driving expression in skin and neurons did not rescue macrophages or microglia in *irf8* mutants (Fig. 5). Interestingly, none of the *irf8* expression constructs appeared to affect the overall number and distribution of macrophages and microglia in wildtype and *irf8* heterozygotes (Fig. 5 and data not shown). These experiments suggest that both immature myeloid and neutrophil populations in *irf8* mutants are capable of developing into mature macrophages after expressing wildtype *irf8*.

## Discussion

**Fig 6:**
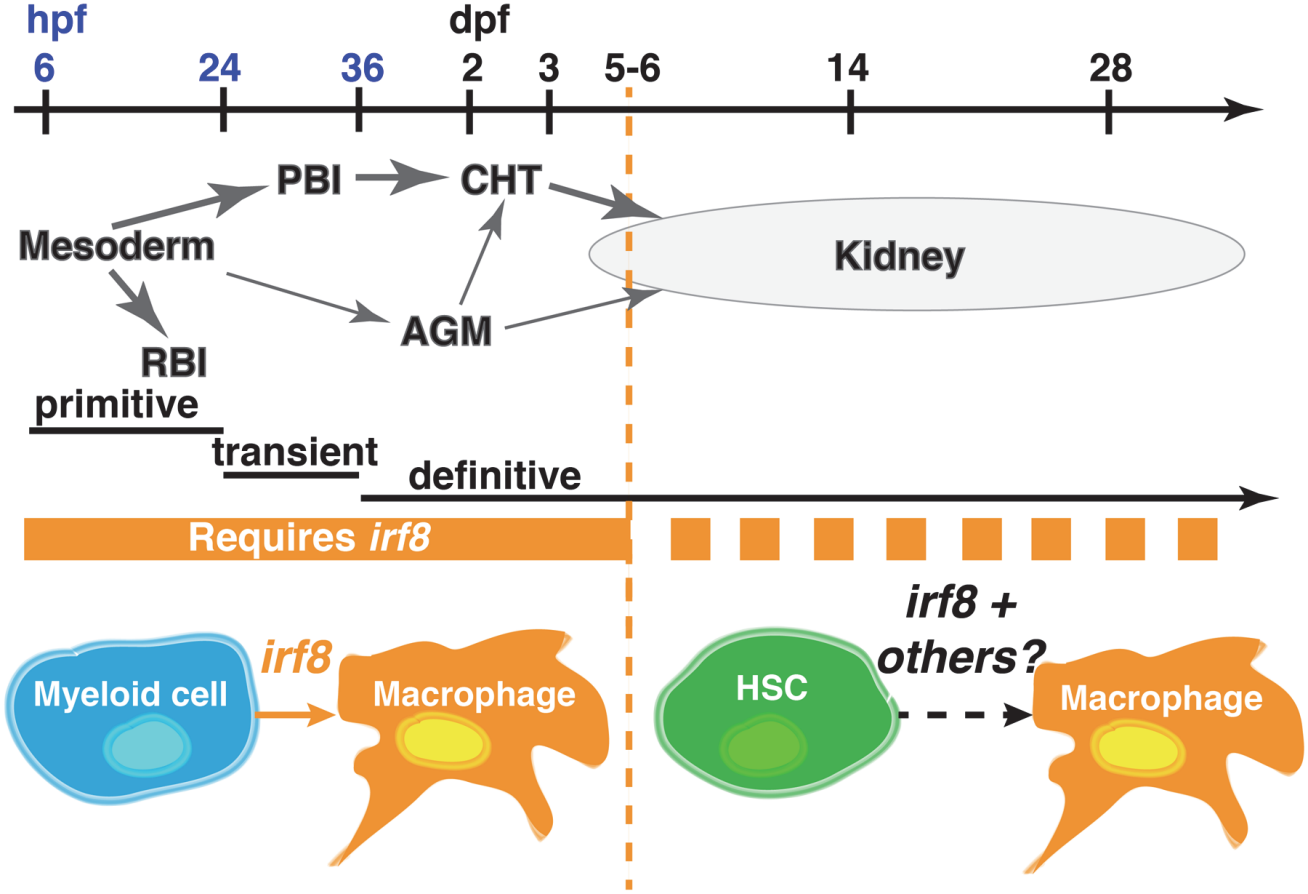
Summary model for role of *irf8* in macrophage ontogeny in zebrafish. *irf8* is required for development of all macrophages in the early developmental period until 5–6 dpf (solid orange bar). Coinciding with the onset of the adult phase hematopoiesis in the kidney starting at ~7 dpf, hematopoiesis relies on *irf8* and other factors for macrophage specification and differentiation (dotted orange bar). RBI, rostral blood island; PBI, posterior blood island; CHT, caudal hematopoietic tissue; AGM, aorta-gonad-mesonephros analog; hpf, hours postfertilization; dpf, days postfertilization.

Our study reveals that specification of all macrophages requires *irf8* during a discrete early period of zebrafish hematopoiesis that includes the primitive and transient definitive waves in zebrafish (Fig. 6). The macrophage deficiency and overproduction of neutrophils and progenitor cells in zebrafish *irf8* mutants are similar to the phenotypes of humans and mice carrying loss-of-function mutations in Irf8 [10–12, 30]. Also similar to Irf8 knockout mice and human patients homozygous for inactivating mutations of Irf8 [11, 12], zebrafish *irf8* null mutants were viable. Zebrafish *irf8* mutants thereby provide a new model to understand the diverse roles of *irf8* in development and innate immunity that will likely be relevant to mammalian Irf8.

Our analysis underscores different genetic controls of hematopoiesis at early and late stages. Despite the complete absence of macrophages in *irf8* mutants at early stages, some mature macrophages were present in *irf8* mutants by mid-larval stages. The recovery of macrophages in *irf8* mutants is more prominent in the head than in the tail through one month of age. This suggests that the late-emerging macrophages in *irf8* mutants may be derived from hematopoietic stem cells (HSCs) in the developing kidney, which is located near the ventral head. The different origins of the macrophages at different stages (primitive myeloid cells early and HSCs later) may explain their differential dependence on *irf8* (Fig. 6). Kidney-derived HSCs may utilize a combination of *irf8* and other factors to specify macrophages, whereas the early myeloid progenitors in the embryo all require *irf8* to develop as macrophages, possibly reflecting a more homogenous population that requires the same genetic program for macrophage differentiation at early stages.

Our data in combination with the analysis of *irf8* morphants [17] provide evidence that *irf8* is essential to regulate granulocyte-macrophage fate decisions in myeloid progenitors and maturation of macrophage precursors. This is consistent with recent characterization of IRF8-EGFP reporter mice that shows strong upregulation of IRF8 reporter expression in granulocyte-myeloid progenitors and monocytic/macrophage progenitors, but low expression in granulocytes [13]. We show that driving expression of *irf8* in the macrophage and neutrophil lineages in *irf8* mutants is sufficient to restore macrophage fate. Similarly, in mouse, induced Irf8 expression in Irf8^-/-^ myeloid progenitors *in vitro* allowed their differentiation into mature macrophages [10]. Although *irf8* expression in neutrophils does restore some macrophages in *irf8* mutants, many excess neutrophils were not converted to macrophages. This likely reflects, in part, the mosaic expression of *irf8* in these assays, but it is also possible that some of the excess neutrophils arise as a secondary consequence of the loss of macrophages, increased myeloid cell death, or both, and not from alterations of progenitor cell fates. Thus, some excess neutrophils may not be competent to convert to macrophages. In mammals, macrophage depletion can cause neutrophilia, possibly due to defective clearance of apoptotic cells after macrophage loss [31], and wounds depleted of macrophages are populated by large numbers of neutrophils [32].

The conservation of Irf8 in macrophage/monocyte specification seems clear, although the extent of the Irf8 requirement appears more far-reaching in zebrafish than in mammals. All macrophages and microglia are eliminated in the *irf8* mutant zebrafish embryo, whereas *Irf8* deficient mice lose a subset of macrophages/monocytes and dendritic cells at embryonic and adult stages [14, 33–36]. This includes a severe but incomplete reduction of the yolksac c-kit+ erthryomyeloid precursors that give rise to microglia [27]. Mouse Irf8 mutants also retain varied levels of other tissue macrophages including Langerhans cells [36]. At the time of macrophage recovery, zebrafish *irf8* mutants do not appear to lack specific subpopulations of macrophages based on anatomical distribution, with the apparent exception of microglia. However, we cannot exclude the possibility that microglia arise at later stages, or that other subsets of macrophage/monocyte are lacking in zebrafish *irf8* mutants.

In summary, we show that Irf8 is essential for the development of all macrophage fates through the early waves of zebrafish hematopoiesis (primitive and transient definitive), but not adult-phase hematopoiesis (Fig. 6). Although the nature of its function changes over time, Irf8 is continuously required to allow normal production of macrophages in zebrafish. Besides its role in myeloid development, Irf8 has essential roles in host defense [37–39]. The generation of zebrafish *irf8* mutants thus provides a new *in vivo* model to dissect the different functions of Irf8 in hematopoiesis and host defense.

## Materials and Methods

### Zebrafish lines and embryos

Embryos from wildtype (TL, AB/TU, and WIK), transgenic (*Tg(lyz*:*EGFP)*[28], *Tg(mpeg1*:*EGFP)*[29], and *Tg(pu*.*1*:*Gal4-UAS-EGFP)*[22]), and *irf8* ^*st95*^ and *irf8* ^*st96*^ heterozygous intercross backgrounds were raised at 28.5°C, and staged by established standards [40]. Embryos were treated with 0.003% 1-phenyl-2-thiourea (PTU) in methylene blue embryo water to inhibit pigmentation. For in situ hybridization and TUNEL staining, zebrafish embryos and larvae were fixed immediately at the indicated time points of analysis using 4% paraformaldehyde/PBS for overnight fixation at 4°C. For fluorescent imaging, zebrafish embryos and larvae up to 7 dpf were imaged in the living animals at the time of analysis, and older larvae up to 31 dpf were imaged after fixation; they were briefly anesthetized using 0.02% MS-222 (tricaine) prior to overnight fixation in 4% paraformaldehyde/PBS. All euthanasia and procedures followed the protocols approved by the Stanford Institutional Animal Care and Use Committee.

### TALEN-targeting to create *irf8* mutations

A pair of transcription activator-like effector nucleases (TALENs) was designed to target a 5′ region of *irf8* near the translational start site that encompasses a unique AvaI restriction site (see Fig. 1A). The web tool TAL Effector-Nucleotide Targeter 2.0 (TALE-NT 2.0; https://tale-nt.cac.cornell.edu) was used to design the TALENs. Recognition sequences for the left and right TALENs are, respectively: 5′-TGAAGTAAAGGTCTACAAGA-3′ and 5′-TATAAGCCACTGTTTCAGTC-3′. The PCR/Golden Gate cloning protocol for creating the TALENs was used [41]. 400–800 pg of TALE nuclease mRNA (transcribed by Sp6 mMessage mMachine Kit, Ambion) was injected into 1–2 cell stage embryos and raised at 28.5°C. To identify founders, injected fish were crossed and a subset of the F1 progeny was assayed for the presence of a mutation in the *irf8* locus based on disruption of the AvaI site; when a mutation was identified the remaining progeny were raised to adulthood. Individual F1 adults carrying an *irf8* mutation disrupting the AvaI site were further analyzed by sequencing, which enabled identification of two specific alleles, *st95* and *st96*, that contain frameshift mutations. To genotype *st95* and *st96*, a PCR fragment of *irf8* exon1 was amplified using the following primers: 5′-ACATAAGGCGTAGAGATTGGACG-3′ and 5′-GAAACATAGTGCGGTCCTCATCC-3′, followed by a digest with AvaI on the PCR product.

### Neutral red assay

For phenotypic analyses of microglia in live fish, neutral red assay was used. 3–6 dpf larvae were incubated in embryo water containing 2.5 μg/ml neutral red at 28.5°C for 2–3 hours, followed by 1–2 water changes, and then analyzed 0.5–24 hours later using a dissecting microscope.

### Whole mount RNA in situ hybridization, TUNEL assay and immunostaining

In situ hybridization on whole zebrafish embryos and larvae from 20 hpf to 3 dpf was performed using standard methods. Antisense riboprobes used were: *lyz*, *mfap4*, and *apoeb* (or *apoe*), as described [21]. TUNEL assay (In situ Cell Death Detection Kit, TMR Red, Roche) was performed as previously described [21]. Subsequent immunostaining was performed using the anti-L-plastin (LCP) antibody [42] at 1:250–500 dilution, followed by DAPI staining. For the cell death analysis, 5 dpf larvae from *irf8* heterozygous intercrosses were processed for TUNEL and L-plastin immunostaining, and genotyped. Images of the sibling and mutant larvae were taken from the same anatomical region caudal to the end of yolk extension on an upright Zeiss Axio Imager.M2 microscope using the 20x (NA 0.8) objective. Most TUNEL signals were concentrated in the CHT of the larval tail; the images were cropped to analyze TUNEL signals only in the CHT using ImageJ. The anatomical region is illustrated in Fig. 3 diagram. Similar threshold levels were set for all images and adjusted to accurately reflect the TUNEL signals to measure area of the nuclei labeled by TUNEL (Fig. 3C,D). Representative tracing of the TUNEL signals to determine area of each labeled nucleus is shown in Fig. 3C,D below the actual fluorescent image of the TUNEL staining. Total number and number of small and other sized TUNEL-positive cells were quantified.

### Time-lapse and fluorescent imaging

For live imaging, embryos were embedded in 1.5% low melting point agarose on glass slides. Fluorescent images were taken on a spinning-disk confocal microscope (Perkin Elmer UltraVIEW VoX imaging system using the Zeiss Axio Observer Z.1 microscope base with the Yokogawa CSU-X1 scanner unit and Hamamatsu EM-CCD C9100–13 camera), unless noted otherwise. The Volocity Acquisition suite was used for 3-dimensional multichannel recording. The following objectives were used: EC Plan-NeoFluar 10x/0.3 Ph1 DIC, Plan-Apochromat 25x/0.8W, and C-Aprochromat 63x/1.2W Korr. Images were analyzed using the Volocity software and ImageJ, and processed using Adobe Photoshop CS5. Static images of live and fixed larvae for analysis of macrophage numbers in Fig. 4 were taken on a Zeiss LSM 5 Pascal confocal microscope using the 20x (NA 0.75) objective. ImageJ Cell Counter tool was used to count the number of cells.

### Cryosectioning for brain microglia analysis

Larvae were fixed overnight in 4% paraformaldehyde/PBS and washed three times in PBS with 0.1% tween. Post-fixed larvae were equilibrated to 30% sucrose, embedded in O.C.T. medium, and snap frozen in dry ice-ethanol bath. Samples were sectioned at 12–14 μm in the head region, and subsequently imaged on an upright Zeiss Axio Imager.M2 microscope using the 10x/0.45 and 40x/0.95 objectives.

### Expression constructs and embryo injection

Full-length *irf8* coding sequence (NM_001002622) was cloned from 5 dpf wildtype cDNA into pCR8/GW/TOPO and verified by sequencing. Tissue-specific expression vectors (*mpeg1*:*irf8; lyz*:*irf8; huc*:*irf8; krt4*:*irf8*) were assembled using multisite Gateway methods [43] in a destination vector containing Tol2-transposon sequences for genomic integration, and *cmlc2-EGFP* for selection. Each plasmid expressing a transgene was co-injected at 12–25 pg along with 50–100 pg of Tol2 transposase mRNA (transcribed by Sp6 mMessage mMachine Kit, Ambion) at 1–4 cell stage. Injected embryos were selected for further analysis based on strong expression of GFP in the heart by the cardiac reporter *cmlc2*:*EGFP*.

## Supporting Information

S1 Fig Kaplan-Meier plot showing survival of homozygous *irf8* mutant zebrafish into adulthood past 3 months of age. A survival study of the progeny from heterozygous *irf8* intercross showed survival of all wildtype *irf8* ^*+/+*^ progenies (n = 10/10), nearly all heterozygous *irf8* ^*+/-*^ fish (n = 16/17), and some homozygous *irf8* ^*-/-*^ mutants (n = 3/5) at 100 dpf. The genotypes of the fish were determined by fin clip assay at 30 dpf, and survival was monitored thereafter, until 100 dpf. Between these stages, 60% of the homozygous mutants survived in this analysis.
(TIFF)

S2 Fig Thin brain sections reveal many microglial cells in wildtype but not in *irf8* mutants, which appear to have only macrophages at 31 dpf. (A) The midbrain optic tectum, where many microglia normally reside, was the region of analysis. (B) Higher magnification of the brain region indicated in dotted box in A. 12–14 um cryosections were taken from wildtype *irf8* ^*+/+*^ and heterozygous *irf8* ^*st95/+*^ fish (n = 4) to compare with *irf8* ^*st95/st9*^ mutants (n = 3) at 31 dpf. Immunostaining for L-plasin (with DAPI as a counterstain) was used to identify macrophages in relation to all cell bodies in the sections. Microglia were identified by their L-plastin expression, elaborate morphology with fine processes, and location in the parenchyma (white arrows). Cells in or adjacent to the interstitial space, vasculature, and ventricular zone were likely macrophages. Autofluorescence in the green channel helped to define the vasculature and distinguish actual L-plastin signal from vasculature autofluorescence in the red channel. *irf8* mutants had a few L-plastin positive cells in the interstitial region and near the ventricular zone that lack fine processes (blue arrows), suggesting that these cells were not microglia. OT, midbrain optic tectum; DC, diencephalon. All scale bars are 50 um.
(TIFF)
