# Supplementary figures and images for: Differential Requirement for irf8 in Formation of Embryonic and Adult Macrophages in Zebrafish
Source: PLoS One. 2015 Jan 23;10(1):e0117513. doi: 10.1371/journal.pone.0117513 (PMC4304715; doi:10.1371/journal.pone.0117513)

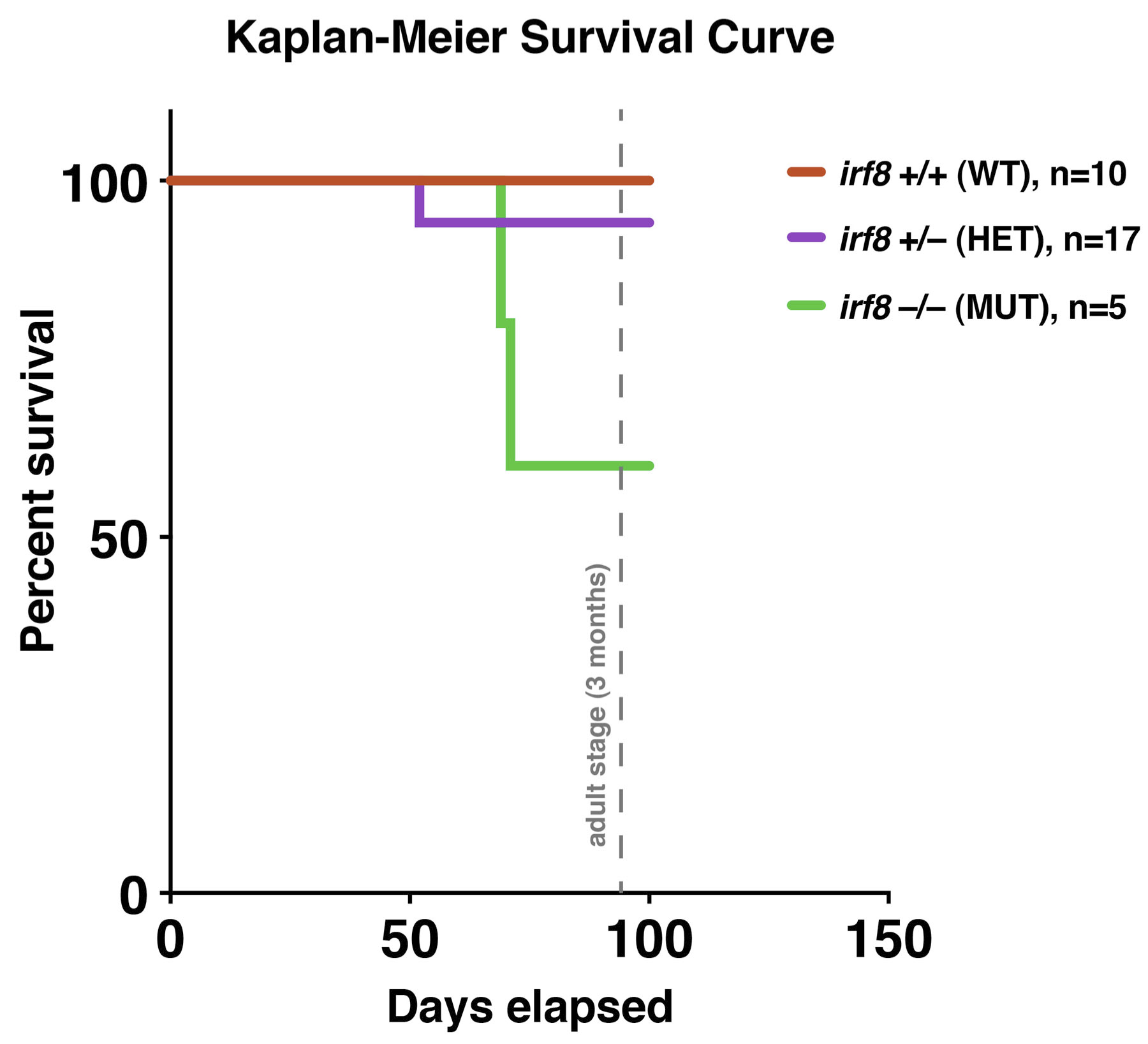

Supplement: S1 Fig — A survival study of the progeny from heterozygous irf8 intercross showed survival of all wildtype irf8 +/+ progenies (n = 10/10), nearly all heterozygous irf8 +/- fish (n = 16/17), and some homozygous irf8 -/- mutants (n = 3/5) at 100 dpf. The genotypes of the fish were determined by fin clip assay at 30 dpf, and survival was monitored thereafter, until 100 dpf. Between these stages, 60% of the homozygous mutants survived in this analysis. (TIFF) [file pone.0117513.s001.tiff]

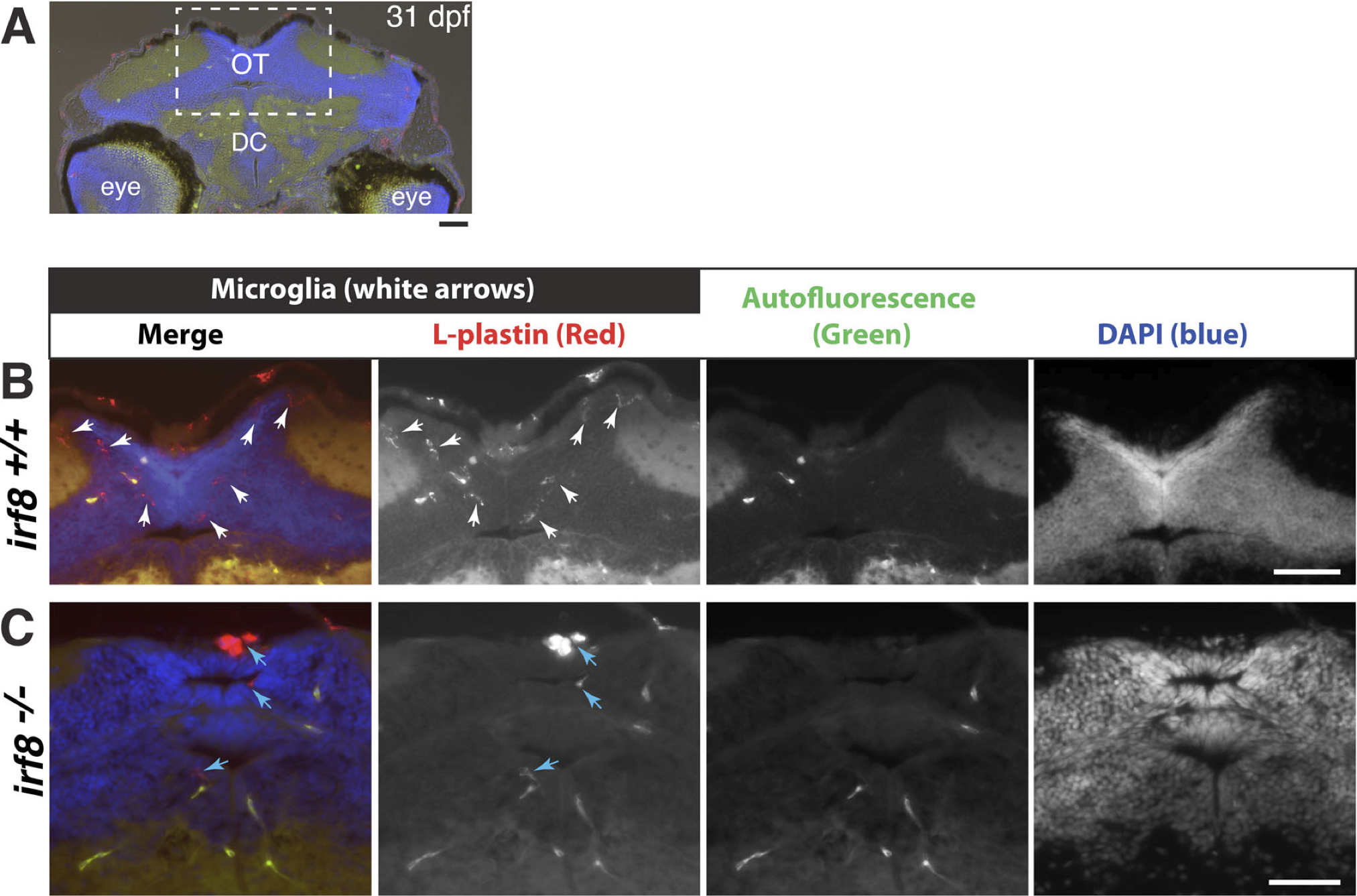

Supplement: S2 Fig — (A) The midbrain optic tectum, where many microglia normally reside, was the region of analysis. (B) Higher magnification of the brain region indicated in dotted box in A. 12–14 um cryosections were taken from wildtype irf8 +/+ and heterozygous irf8 st95/+ fish (n = 4) to compare with irf8 st95/st9 mutants (n = 3) at 31 dpf. Immunostaining for L-plasin (with DAPI as a counterstain) was used to identify macrophages in relation to all cell bodies in the sections. Microglia were identified by their L-plastin expression, elaborate morphology with fine processes, and location in the parenchyma (white arrows). Cells in or adjacent to the interstitial space, vasculature, and ventricular zone were likely macrophages. Autofluorescence in the green channel helped to define the vasculature and distinguish actual L-plastin signal from vasculature autofluorescence in the red channel. irf8 mutants had a few L-plastin positive cells in the interstitial region and near the ventricular zone that lack fine processes (blue arrows), suggesting that these cells were not microglia. OT, midbrain optic tectum; DC, diencephalon. All scale bars are 50 um. (TIFF) [file pone.0117513.s002.tiff]
